# Development of [^131^I]I-EOE-TPZ and [^131^I]I-EOE-TPZMO: Novel Tirapazamine (TPZ)-Based Radioiodinated Pharmaceuticals for Application in Theranostic Management of Hypoxia

**DOI:** 10.3390/ph12010003

**Published:** 2019-01-01

**Authors:** Hassan Elsaidi, Fatemeh Ahmadi, Leonard I. Wiebe, Piyush Kumar

**Affiliations:** 1 Department of Oncology, Cross Cancer Institute, Faculty of Medicine and Dentistry, University of Alberta, 11560 University Avenue, Edmonton, AB T6G 1Z2, Canada; elsaidi@ualberta.ca (H.E.); leonard.wiebe@ualberta.ca (L.I.W.); 2 Department of Pharmaceutical Chemistry, Faculty of Pharmacy, University of Alexandria, El Sultan Hussein St. Azarita, Alexandria 21521, Egypt; 3 Current address: Centre for Probe Development and Commercialization, 1280 Main Street West, Hamilton, ON L8S 4K1, Canada; fahmadi@ualberta.ca; 4 Joint Appointment to Faculty of Pharmacy and Pharmaceutical Sciences, University of Alberta, Edmonton, AB T6G 2E1, Canada

**Keywords:** hypoxia, radiosensitizer, benzotriazine-1,4-dioxide (BTDO), benzotriazine-1-monoxide (BTMO), tirapazamine (TPZ), SR 4317, radioiodination

## Abstract

*Introduction*: Benzotriazine-1,4-dioxides (BTDOs) such as tirapazamine (TPZ) and its derivatives act as radiosensitizers of hypoxic tissues. The benzotriazine-1-monoxide (BTMO) metabolite (SR 4317, TPZMO) of TPZ also has radiosensitizing properties, and via unknown mechanisms, is a potent enhancer of the radiosensitizing effects of TPZ. Unlike their 2-nitroimidazole radiosensitizer counterparts, radiolabeled benzotriazine oxides have not been used as radiopharmaceuticals for diagnostic imaging or molecular radiotherapy (MRT) of hypoxia. The radioiodination chemistry for preparing model radioiodinated BTDOs and BTMOs is now reported. *Hypothesis*: Radioiodinated 3-(2-iodoethoxyethyl)-amino-1,2,4-benzotriazine-1,4-dioxide (I-EOE-TPZ), a novel bioisosteric analogue of TPZ, and 3-(2-iodoethoxyethyl)-amino-1,2,4-benzotriazine-1-oxide (I-EOE-TPZMO), its monoxide analogue, are candidates for in vivo and in vitro investigations of biochemical mechanisms in pathologies that develop hypoxic microenvironments. In theory, both radiotracers can be prepared from the same precursors. *Methods*: Radioiodination procedures were based on classical nucleophilic [^131^I]iodide substitution on Tos-EOE-TPZ (P1) and by [^131^I]iodide exchange on I-EOE-TPZ (P2). Reaction parameters, including temperature, reaction time, solvent and the influence of pivalic acid on products’ formation and the corresponding radiochemical yields (RCY) were investigated. *Results*: The [^131^I]iodide labeling reactions invariably led to the synthesis of both products, but with careful manipulation of conditions the preferred product could be recovered as the major product. Radioiodide exchange on P2 in ACN at 80 ± 5 °C for 30 min afforded the highest RCY, 89%, of [^131^I]I-EOE-TPZ, which upon solid phase purification on an alumina cartridge gave 60% yield of the product with over 97% of radiochemical purity. Similarly, radioiodide exchange on P2 in ACN at 50 ± 5 °C for 30 min with pivalic acid afforded the highest yield, 92%, of [^131^I]I-EOE-TPZMO exclusively with no trace of [^131^I]I-EOE-TPZ. In both cases, extended reaction times and/or elevated temperatures resulted in the formation of at least two additional radioactive reaction products. *Conclusions*: Radioiodination of P1 and P2 with [^131^I]iodide leads to the facile formation of [^131^I]I-EOE-TPZMO. At 80 °C and short reaction times, the facile reduction of the N-4-oxide moiety was minimized to afford acceptable radiochemical yields of [^131^I]I-EOE-TPZ from either precursor. Regeneration of [^131^I]I-EOE-TPZ from [^131^I]I-EOE-TPZMO is impractical after reaction work-up.

## 1. Introduction

Solid tumors frequently demonstrate rapid growth and aberrant vasculature, leading to microenvironmental deficiencies of oxygen (hypoxia), nutrients and therapeutic drugs. Tumor hypoxia is an early event and an independent risk factor for progression of all types of cancers. Hypoxic tumors are relatively more resistant than oxygenated tumors to killing by ionizing radiation during conventional radiotherapy, and recurrent cancers may be metastatically aggressive. Clearly, identification of hypoxic microenvironments and more effective cancer management based on assessments of their hypoxic stature is central to effective treatment [1,2,3,4,5]. In vivo imaging offers insights into the detection and management of tumor hypoxia [6,7,8,9,10] and, of the imaging modalities available, nuclear imaging is both effective and widely available. Radiolabeled azomycin (2-nitroimidazole) derivatives are the most studied and used radiopharmaceuticals for hypoxia imaging [11,12,13].

Like the nitroimidazoles, 1,2,4-benzotriazines (BTDOs such as 1,2,4-benzotriazine-3-amino-1,4-dioxide (tirapazamine, TPZ) and its derivatives are bioactivated via a single electron reduction that is reversible in the presence of oxygen [14]. TPZ was synthesized as a potential antimicrobial agent in 1957 [15,16], and the rediscovery of TPZ and its monoxide homologue (TPZMO; SR 4317) as radiosensitizers [17,18] opened the door to hypoxic radiosensitization by bioreductivately-activated BTDOs that offer an alternative mechanism to that of the nitroimidazoles [19,20,21,22]. Radiolabeled BTDOs and BTMOs for hypoxia imaging or radiotherapy appear unreported to date.

**Figure 1:**
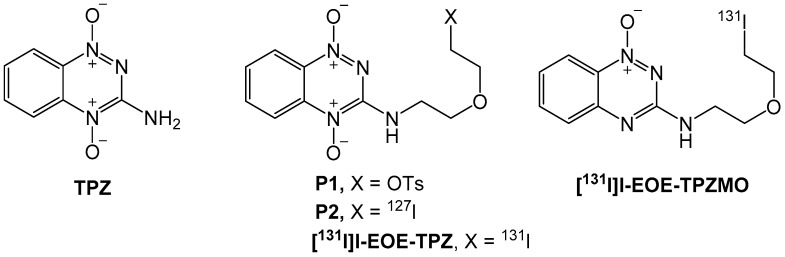
Structures of **TPZ** (tirapazamine), **P1** (Tos-EOE-TPZ; X = tosyl), **P2** (I-EOE-TPZ; X = I = ^127^I), and [^131^I]I-EOE-TPZMO.

A recent publication on the synthesis of 3-(2-iodoethoxyethyl)-amino-1,2,4-benzotriazine-1,4-dioxide (I-EOE-TPZ) and 3-(2-iodoethoxyethyl)-amino-1,2,4-benzotriazine-1-monoxide (I-EOE-TPZMO) reports that the in vitro toxicity and radiosensitizing potency of I-EOE-TPZ were similar to that of TPZ [23]. These investigations into the radiosyntheses of [^131^I]I-EOE-TPZ and its monoxide homologue, [^131^I]I-EOE-TPZMO, represent the next step in the evaluation of these compounds as radiotheranostic pharmaceuticals. The chemical structures of TPZ, Tos-EOE-TPZ, the precursor for nucleophilic radioiodination (P1), and I-EOE-TPZ, the precursor for isotope exchange radiolabeling (P2), and I-EOE-TPZMO are depicted in Figure 1.

## 2. Results

[^131^I]I-EOE-TPZ and [^131^I]I-EOE-TPZMO, the desired products, were both identified in reaction mixtures following nucleophilic substitution of the tosylate precursor (**P1**) by [^131^I]iodide, and upon halogen isotope exchange between [^131^I]iodide and non-radioactive precursor I-EOE-TPZ (**P2**). Radiochemical yields were strongly dependent upon reaction conditions.

### 2.1. Nucleophilic Radioiodination of P1

**Figure 2:**
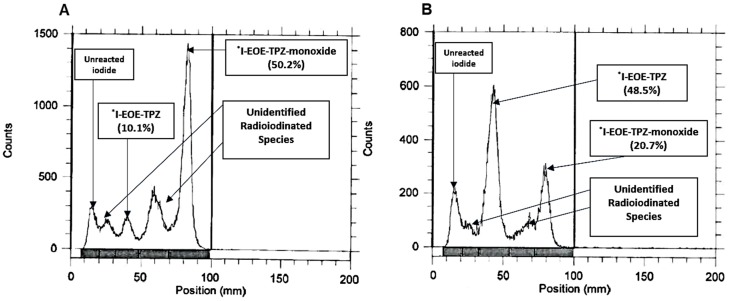
RTLC of the reaction mixture post-nucleophilic radioiodination of **P1** by [^131^I]iodide in ACN after 60 min at 100 °C (**A**) and at 80 °C (**B**).

Nucleophilic reactions on P1, irrespective of reaction duration, in either DMF or ACN at 22 °C and at 60 °C demonstrated no formation of either [^131^I]I-EOE-TPZ or [^131^I]I-EOE-TPZMO, with only unreacted [^131^I]iodide detected by RTLC (Table 1). However, reaction for 60 min at higher temperatures (80 °C and 100 °C) in ACN afforded both products. Using DMF as a solvent at these (higher) temperatures also led to the formation of *I-EOE-TPZ, albeit in lower yields. Radioiodination at 80 °C for 60 min in ACN appeared to be the best condition for synthesizing [^131^I]I-EOE-TPZ (RCY 48.5%; entry 3 Table 1) via nucleophilic substitution of precursor P1; at 100 °C, this reaction favored production of [^131^I]I-EOE-TPZMO (RCY 50.2%; entry 4 Table 1). Reaction conditions and product yields are given in Table 1 and typical radiochromatograms for 80 and 100 °C are shown in Figure 2 (A and B).

### 2.2. Isotope Exchange Radioiodination of P2

**Figure 3:**
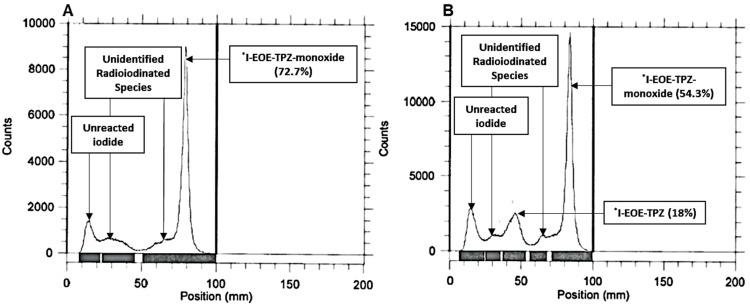
RTLC of the reaction mixture following [^131^I]iodide exchange on **P2** at 22 °C. [^131^I]I-EOE-TPZMO is the main product by 30 min (**A**), [^131^I]I-EOE-TPZ is evident in low radiochemical yield (18%) at 60 min (**B**).

Radioiodination of I-EOE-TPZ (**P2**) by [^131^I]iodide was monitored under a range of reaction conditions. The first attempt, using the conditions applied to nucleophilic labelling (**P1)** in DMF at 22 °C, showed a high yield of [^131^I]I-EOE-TPZMO and no [^131^I]I-EOE-TPZ at 30 min, and a small yield of [^131^I]I-EOE-TPZ (18%) at 1 h; both radiochromatograms revealed a complex mixture of the desired products, radioiodide and at least two unknown radioiodinated by-products (Figure 3 and Table 2).

**Figure 4:**
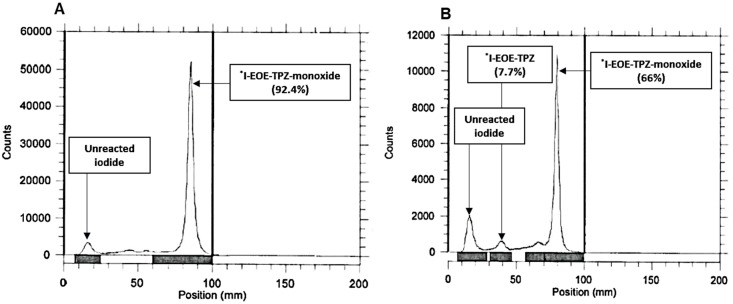
RTLC of the post-labelling exchange reaction mixture using precursor **P2** in ACN and pivalic acid after 30 min (**A**) and 60 min (**B**) at 50 °C.

Exchange radioiodination of P2 was conducted in ACN at 50 °C in the presence of pivalic acid for 30 min. Under these conditions, RTLC showed the highest yield, 92.4%, of [^131^I]I-EOE-TPZMO but with no [^131^I]I-EOE-TPZ (Figure 4A). At 60 min, a third radioactive compound at *R_f_* 0.75, running just behind [^131^I]I-EOE-TPZMO, was apparent in the RTLC as well as a trace evidence, 7.7%, of [^131^I]I-EOE-TPZ (Figure 4B).

**Figure 5:**
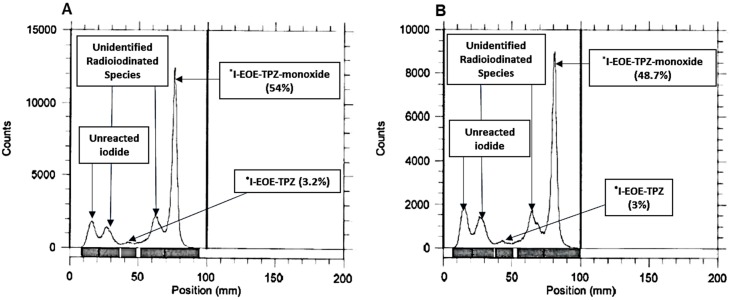
RTLC of the exchange reaction mixture using precursor **P2** and pivalic acid in ACN after 30 min (**A**) and 90 min (**B**) at 22 °C.

To determine if pivalic acid was responsible for promoting the formation of the peak at *R_f_* 0.75 at the higher temperature (i.e., 50 °C), radioiodination was performed at a room temperature (22 °C) after 30, 60 and 90 min in ACN. The RTLCs showed an increased appearance in by-products at *R_f_* 0.35 and 0.75 (Figure 5). A room temperature experiment using pivalic acid in a protic solvent (ACN/ETOH mixture) yielded similar (no improvement) results, but with lower peak resolution (not shown).

**Figure 6:**
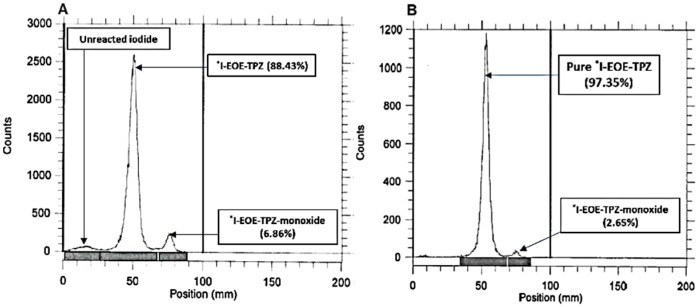
(**A**) The RTLC of the exchange-labeled [^131^I]I-EOE-TPZ (**P2**) reaction mixture in ACN, 30 min after reacting at 80 °C with sodium [^131^I]iodide. (**B**) The RTLC of (**A**) after purification through a neutral alumina cartridge.

Radioiodine exchange on precursor P2 in ACN at 80 °C for 30 min provided the highest radiochemical yield of [^131^I]I-EOE-TPZ (89%; Table 2). Subsequent solid phase purification on an alumina cartilage afforded [^131^I]I-EOE-TPZ in radiochemical yields of 60% with over 97% radiochemical purity and a trace of [^131^I]I-EOE-TPZMO (Figure 6).

## 3. Discussion

The radiosynthesis of [^131^I]I-EOE-TPZ by nucleophilic radioiodination of the corresponding tosylate, Tos-EOE-TPZ, was associated with the production of a second hypoxia-selective radiosensitizer, [^131^I]I-EOE-TPZMO. In fact, minimizing the production of this radiolabeled 1-monoxide was a major challenge to preparing the radiolabeled, fully oxidized, 1,4-dioxide, analogue. There is precedence to the formation of the 1-monoxide in the literature, both as SR 4317, an analogue of TPZ [16,24], and as a product of TPZ metabolism [25,26]. The synthetic chemistry of the X-EOE-TPZ series similarly involved the production of the corresponding X-EOE-TPZMO 1-oxides [23]. Unfortunately, up-oxidation of the monoxides to their 1,4-dioxides was difficult or even unachievable, thereby squelching the idea of simply using the highly efficient production of [^131^I]I-EOE-TPZMO (by either halogen exchange or nucleophilic substitution) and then simply oxidizing the monoxide to [^131^I]I-EOE-TPZ.

The unanticipated, but facile reduction of I-EOE-TPZ by (radio)iodide in both exchange labelling and nucleophilic substitution procedures appears to lie in the ease of oxidizing iodide to iodonium species. Iodide may first serve as the single electron donor, giving rise to the BTDO radical intermediate, and also provide the second electron to form the monoxide. Examples of functional group reductions via iodide salts include amine N-oxides [27], isoxazolodines [28], sulfoxides [29] and graphene oxide [30] and others [31] Plausible mechanisms have been proffered for the bioreduction of TPZ [24,25,26] to its 1-monoxide, and these may apply when iodide is the initial electron donor.

**Scheme 1:**
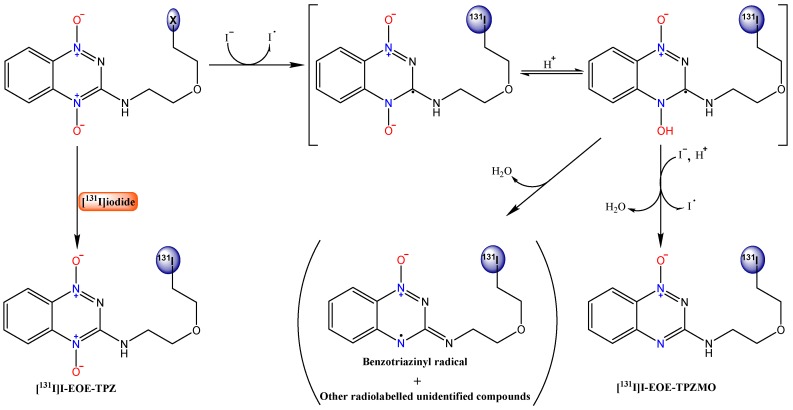
A proposed model for the formation of [^131^I]I-EOE-TPZMO by [^131^I]iodide during isotope exchange with I-EOE-TPZ and nucleophilic substitution on Tos-EOE-TPZ. This model is based in part on a model proposed by Siim et al. for TPZ metabolism [26].

Although it seems unlikely that pivalic acid directly affects the initial single electron reduction in this scheme, facilitation of radioiodide/halogen exchange radiolabeling by pivalic acid is well established in the radiopharmaceutical literature [32] In the current case, it is possible that pivalic acid skews the equilibrium between the protonated and non-protonated radical in the aprotic solvent, an effect that may not influence formation of the reduced monoxide, but that leads to the formation of other reductive intermediates as well. The latter postulate is supported by the prominence of two new radioactive by-products in the pivalic acid isotope exchange reactions, products that are also prominent in the nucleophilic substitution reactions at 100 °C and when the reaction is carried out in a protic (ACN-ethanol) solvent. A plausible mechanism for the facile conversion of I-EOE-TPZ and Tos-EOE-TPZ to [^131^I]I-EOE-TPZMO, is presented in Scheme 1.

## 4. Experimental

### 4.1. Materials

Precursors P1 and P2 were synthesized as reported [23] Anhydrous EtOH, sterile water for injection (SWFI) and 0.9% bacteriostatic saline were purchased from commercial suppliers. All solvents (ethyl acetate [EtOAc], acetonitrile [ACN] and dimethylformamide [DMF] were reagent grade, purchased from commercial suppliers, and used without further purification. Sodium [^131^I]iodide was purchased by the Edmonton Radiopharmacy Center from NTP Radioisotopes (Pretoria, South Africa), and was provided to us at no cost for experimental development. Reacti-V-vials (4 mL; reactivial) were purchased from Wheaton, Millville, NJ, USA, while disposable sterile items (syringes and needles of various size, vent needles, product vials [Hollister, 20 mL], GS filters [22 µm pore; Millipore, Cork, Ireland], neutral alumina cartridges [Waters, Milford, MS, USA]) were purchased from respective suppliers. Progress of reactions, and radiochemical purity of final products were monitored on silica gel pre-coated glass TLC plates (2.5 × 7.5 cm; Whatman). RadioTLC (RTLC) plates were scanned using a Bioscan TLC scanner (Eckert & Ziegler, Berlin, Germany). Specific quality control tests that included confirming radionuclidic identity by determining the half-life by counting a sample of purified product (from optimized synthesis batch) in a counting well and checking pH of purified product solution using pH test strips. The authentic reference standards, I-EOE-TPZ and I-EOE-TPZMO monoxide that were co-spotted, were visible as red (*R_f_* 0.45 ± 0.05) and yellow (*R_f_* 0.85± 0.05) spots, respectively.

### 4.2. Methods

#### 4.2.1. Nucleophilic Radioiodination of Tos-EOE-TPZ (Precursor P1)

It started by mixing a solution of P1 (100 µg/100 µL) in the selected solvent and adding this to the reactivial containing [^131^I]iodide (nominally 37 kBq). After radiometry, the vial was placed on a pre-heated block and radioiodination was performed for the specified time. The reaction vial was removed, cooled to room temperature, and then aliquots were taken for RTLC. Data are presented in Table 1.

#### 4.2.2. Halogen Isotope Exchange Radioiodination of I-EOE-TPZ (Precursor P2)

The procedure was based on the procedure used for synthesizing P2.^23^ P2 (100 µg), pre-dissolved in an appropriate reaction solvent (100 µL), was added to the reaction v-vial containing sodium [^131^I]iodide (nominally 37 kBq). The vial was capped and the radioactivity measured. Reaction vials that required heating were placed on a heating block (50 or 80 °C). Progress of reactions was monitored using radioTLC and co-chromatography with authentic reference standards. Reaction times, temperatures and product yields for exchange radiolabeling are given in Table 2. In reactions where pivalic acid was used, a solution of P2 (100 µg) in acetonitrile (100 µL) was added to pivalic acid (3.5 mg), the solution was gently swirled and then transferred to a reactivial containing [^131^I]iodide. 

### 4.3. Cartridge-Based Purification

Once the labeling process was complete, the reaction vial was cooled in an ice-bath. Acetonitrile (10 µL) was added to the reaction vial, the vial was gently swirled to dissolve the mixture and the contents were diluted with additional SWFI (10 mL) and then the entire solution was withdrawn into a 20 mL syringe. After removing the syringe needle, the syringe barrel was attached to a Waters alumina cartridge (preconditioned by USP-grade ethanol [10 mL], followed by sterile water [10 mL]) fitted with a filter (Millex GS, Millipore, Cork, Ireland) and needle assembly that was connected to a vented 20 mL sterile product vial. Contents of the needle were slowly pushed through the cartridge to recover the purified products.

## 5. Conclusions

Two approaches to radiolabel I-EOE-TPZ are reported. Both methods, isotope exchange and nucleophilic substitution, produce two compounds of interest, [^131^I]I-EOE-TPZ and [^131^I]I-EOE-TPZMO, their relative proportions being dependent on reaction conditions. [^131^I]I-EOE-TPZMO was obtained almost exclusively in high yield from P2 via isotope radioiodination in ACN and pivalic acid after 30 min at 50 °C. Whereas the highest yield of [^131^I]I-EOE-TPZ was obtained from P1 via halogen isotope exchange radioiodination in ACN and no pivalic acid after 60 min at 80 °C. A simple solid phase extraction process, a methodology that is preferred in clinical settings, was developed and used to purify and isolate [^131^I]I-EOE-TPZ in 45%–60% radiochemical yield and >97% purity. 

This is the first report of developing bioreductively-activated, radiohalogenated BTDO and BTMO molecules for assessing focal hypoxia. Preclinical evaluations of these radiotracers are in progress in animal models of tumor hypoxia.

## Figures and Tables

**Table 1:** Radioiodination of precursor Tos-EOE-TPZ (**P1)** via nucleophilic substitution of tosyl by [^131^I]iodide.

Solvent	Temp (°C)	Time (min)	[^131^I]I-EOE-TPZ % of Total [^131^I]	[^131^I]I-EOE-TPZMO % of Total [^131^I]
ACN	22	30 and 60	0, 0	0, 0
ACN	60	30 and 60	0, 0	17.2, 31.2
ACN	80	60	48.5	20.7
ACN	100	60	10.1	50.2
DMF	22	30 and 90	0, 0	0, 0
DMF DMF DMF	60 80 100	30 and 60 60 60	0, 0 24.6 7.8	0, 0
				16.7
				34.2

**Table 2:** Radioiodination conditions and product yields for [^131^I]iodide exchange on **P2**.

Solvent	Temp (°C)	Time (min)	Pivalic Acid (mg)	[^131^I]I-EOE-TPZ % of Total [^131^I]	[^131^I]I-EOE-TPZMO % of Total [^131^I]
DMF	22	30, 60	0	0, 18	72.7, 54.3
ACN	22	30, 60	0	0, 0	34.7, 7.5
ACN	80	30	0	**89**	6.9
ACN	50	30, 60	3.5	0, 7.7	**92.4**, 66
ACN	22	30, 60, 90	3.5	3.2, 3.5, 3	54, 50, 48.7
EtOH/ACN	22	30, 60, 90	3.5	0, 0, 0	43, 42.4, 42.2
